# Quality of life and psychological distress in women with recurrent miscarriage: a comparative study

**DOI:** 10.1186/s12955-018-0982-z

**Published:** 2018-07-28

**Authors:** Zahra Tavoli, Mahsa Mohammadi, Azadeh Tavoli, Ashraf Moini, Mohammad Effatpanah, Leila Khedmat, Ali Montazeri

**Affiliations:** 1 Department of Obstetrics and Gynecology, School of Medicine, Tehran University of Medical Science, Tehran, Iran; 2 School of Medicine, Tehran University of Medical Sciences, Tehran, Iran; 3 Department of Psychology, Faculty of Educational Sciences and Psychology, Alzahra University, Tehran, Iran; 4 Department of Psychiatry, School of Medicine, Tehran University of Medical Science, Tehran, Iran; 5 Department of Community Medicine, School of Medicine, Tehran University of Medical Science, Tehran, Iran; 6 Population Health Research Group, Health Metrics Research Centre, Iranian Institute for Health Sciences Research, ACECR, Tehran, Iran

**Keywords:** Recurrent miscarriage, Quality of life, Anxiety and depression

## Abstract

**Background:**

This study aimed to evaluate quality of life and psychological distress in Iranian women with recurrent miscarriage and to compare it in women without miscarriage.

**Methods:**

This was a comparative study of quality of life among women with and without recurrent miscarriage. Cases were selected from patients with complain of recurrent miscarriage and comparison group were selected from women attending to two teaching hospitals for annual screening. Quality of life (QOL) was measured using the 36-Item Short Form Survey (SF-36). In addition the Hospital Anxiety and Depression Scale (HADS) were used to measure anxiety and depression. Comparison was made between two groups using the independent samples t-test and chi-square.

**Results:**

In all 105 women with recurrent miscarriage and 105 healthy women were studied. The socio-demographic status for both groups was similar. Women with recurrent miscarriage showed a significant higher degree of psychological distress [mean (SD) anxiety score was: 10.6 (2.3) vs. 9.1 (2.2), *P* < 0.0001; and mean (SD) depression score was: 11.0 (2.3) vs. 9.5 (1.9), *P* < 0.0001]. In addition women with recurrent miscarriage reported significantly lower level of quality of life in all domains (role physical, general health, vitality, social functioning, role emotional, and mental health, all *P* values < 0.0001), except for physical functioning (*P* = 0.06) and bodily pain (*P* = 0.17).

**Conclusion:**

The findings demonstrated that women with recurrent miscarriage reported extensive functional disability, and lower level of well-being compared to women without recurrent miscarriage. The findings have some implications for prenatal care and suggest that appropriate treatment of recurrent miscarriage is essential.

## Background

Recurrent pregnancy loss or recurrent miscarriage is characterized as three or more consecutive pregnancy loss prior to 20 weeks from the last menstrual period. Spontaneous pregnancy loss has been estimated to be prevalent in approximately 15% of clinically diagnosed pregnancies [1]. There are a number of etiological causes for recurrent miscarriage such as immunologic, genetic, and anatomic abnormalities, endocrine disorders, infectious, heritable and/or acquired thrombophilias and environmental factors. However, after the actual evaluation of recurrent pregnancy loss remain unexplained in 60% of cases [2]. Qualitative studies indicated that a history of miscarriage could harm women and be associated with feeling anxious, development of psychological disorders, and affecting quality of life in this population [3].

Most studies on quality of life and psychological disorders come from more developed countires. Women with a history of recurrent miscarriage experience an increase in depressive symptoms and may be at increased risk of negative psychological effects such as pregnancy-related anxiety, depression, irritability, excessive fatigue, fear, sleep disorders and lack of concentration [4, 5] The increase in psychological morbidity immidetly following miscarriage among women are well documented [6].

The importance of psychological factors and socioeconomic status instantly affecting pregnancy or mediating the effects leading to pregnant loss continues to be underestimated in clinical grounds especially recurrent miscarriage, despite research extrapolating their importance. In parallel, health behaviors of women with history of recurrent miscarriage are of great concern. Understanding of women’s health behaviors subsequent to miscarriages is very important for the promotion of optimal health for women with history of recurrent miscarriage. Therefore, the purpose of this study was to evaluate the effects of recurrent miscarriage on quality of life and psychological distress of women with recurrent miscarriage.

## Methods

### Study population and ***data collection***

This was a cross sectional study and participants were selected from 15 to 50-years-old women attending the gynecology outpatient clinics at two teaching hospitals affiliated to Tehran University of Medical Sciences between 2014 and 2015. A sample of women who had a history of three or more recurrent miscarriage (as defined: any pregnancy involuntarily ending before 20 weeks), and a comparison group consisting of a sample of women who did not have recurrent miscarriage, and did not face infertility problems were entered into the study. Exclusion criteria included women with a history of psychiatric disorders, having a history of addiction, treated with anti-anxiety and depression drugs, pregnant at the time of study, and suffering from chronic diseases. All women in both groups were apporached during the study period and they were asked to respond to the study questionnaires. They were assured that their information would remain confidential. Informed consent was obtained from all participants.

### Sample size calculation

The sample size was calculated using the following formula

$$ \mathrm{n}=\frac{{\left({{{\mathrm{z}}_1}_{-}}_{\raisebox{1ex}{$a$}\!\left/ \!\raisebox{-1ex}{$2$}\right.}+{\mathrm{z}}_{1-\beta}\right)}^2\left({\mathrm{s}}_1^2+{\mathrm{s}}_2^2\right)}{{\left({\upmu}_2-{\upmu}_1\right)}^2} $$

As such to have a study with a 80.0% power and able to detect a 20% difference in quality of life score between women with and without miscarriage a sample of 100 women in each group was thought.

### Questionnaires

Data were collected using a demographic questionnaire, the Short form Health survey (SF-36), and the Hosptial Anxiety and Depression Scale (HADS).

1. A demographic questionnaire containing 19 questions was admistered to collect data on age, occupational status, smoking, education, number of children, number of abortions, gestational age at the time of abortion and time of abortion.
2. The SF-36 questionnaires: this is a general measure of health-related quality of life and contains 8 subscales namely physical functioning, role physical, bodily pain, general health, vitality, social functioning, and role emotional and mental health. Score on each subscale range from 0 (the worse) to 100 (the best). Psychometric properties of the Iranian version of the questionnaire are well documented [7].
3. The Hospital Anxiety and Depression Scale (HADS) was used to evaluate the levels of anxiety and depression. The HADS is a fourteen-item scale with two subscales including anxiety and depression and score on each subscale range from 0 to 21 with higher scores indicating higher level of anxiety and depression. Score of 11 or more is considered as a case suffering from disorder that usually requires treatment, scores 8–10 represent the boarderline condition that individuals who have these scores are usually referred for psychiatric assessments, and scores between 0 to 7 show normal statuses [8]. The Iranian version of the HADS exists and its psychometric properties are reported elsewhere [9].

### Statistical analysis

Descrptive statistics were used to explore the data. We performed independent samples t-test and chi-square for group comparison where necessary. *P* value less than 0.05 was considered as significant level.

### Ethics

The ethics committee of Tehran University of Medical; Sciences approved the study. All participants gave informed consent prior to the study commence.

## Results

In all 210 women were entered into the study. The mean age of women with and without miscarriage was 32.1 (SD =4.7) and 32.2 (5.6) years, respectively (*P* = 0.86). Furthermore, 72.4% of participants were housewife and 27.6% were employed. In terms of educational, the majority of women had higher education (53.3%). Overall 52.9% of the participants had a history of childbirth and 47.1% had no successful childbirth history. There were no significant differences between the study groups. However, there was a significant difference in terms of having a child as expected (*P* < 0.0001). The results are shown in Table 1.

**Table 1:** Descriptive information regarding education, occupation and childbirth in women under the study

	With recurrent miscarriage (*n* = 105)	Without recurrent miscarriage (*n* = 105)	
	No. (%)	No. (%)	P*
Age			
Mean (SD)	32.2 (4.7)	32.1 (5.6)	0.86
Education			0.076
Primary	7 (6.7)	2 (1.9)	
Secondary	47 (44.8)	42 (40)	
Higher Education	51 (48.6)	61 (58.1)	
Occupation			0.80
Housewife	76 (72.4)	64 (61)	
Employed	29 (27.6)	41 (39)	
Having a child			0.0001
Yes	26 (24.8)	85 (81)	
No	79 (75.2)	19 (20)	

*All *P* values derived from chi-square test except for age that derived from two independent samples *t*-test

The quality of life data are summarized in Table 2. There were significant differences between women with and without miscarriage in all quality of life subscales as measured by the SF-36 (*P* < 0.0001) except for physical functioning (*P* = 0.06) and bodily pain (*P* = 0.17).

**Table 2:** Comparison of quality of life between women with and without miscarriage

	With recurrent miscarriage (*n* = 105)	Without recurrent miscarriage (*n* = 105)	
	Mean (SD)	Mean (SD)	P*
Physical functioning	73.7 (18.5)	79.1 (23.1)	0.06
Role physical	44.7 (40)	66.1 (37.4)	< 0.0001
Bodily pain	49.6 (12.3)	47.5 (10.1)	0.17
General health	53.6 (17.9)	66.3 (19.2)	< 0.0001
Vitality	50.2 (15.3)	59.7 (15.7)	< 0.0001
Social functioning	58.8 (19.6)	76.6 (22.3)	< 0.0001
Role emotional	40.6 (38.6)	64.7 (39.7)	< 0.0001
Mental health	54.2 (16.2)	66.7 (17.6)	< 0.0001

*Derived from two independent samples *t*-test

Table 3 presents data for anxiety and depression. There were significant differences between women with and without miscarriage indicating that women with recurrent miscarriage experienced higher levels of anxiety and depression.

**Table 3:** Comparison of anxiety and depression in women with and without recurrent miscarriage

	With recurrent miscarriage (*n* = 105)	Without recurrent miscarriage (*n* = 105)	
	Mean (SD)	Mean (SD)	P*
Anxiety	9.1 (2.2)	10.6 (2.3)	0.0001
Depression	11.0 (2.3)	9.5 (1.9)	< 0.0001

*Derived from independent two samples *t*-test

Furthermore, the data demonstrated that there were no significant differences in quality of life score between women with and without child in recurrent miscarriage group except for general health (*P* = 0.001) and mental health (*P* = 0.03). In addition, when anxiety and depression was compared between these women the findings showed that those women with recurrent micarriage who did not have a child were suffering more comapred to those who had at least one child (*P* value for anxiety = 0.005, *P* value for depression = 0.02). The findings are shown in Table 4.

**Table 4:** Comparison of anxiety, depression and quality of life among women with a history of recurrent miscarriage with or without child

	Women with recurrent miscarriage with child (*n* = 26)	Women with recurrent miscarriage without child (*n* = 79)	
	Mean(SD)	Mean(SD)	P*
Anxiety	9.57 (2.3)	11.0 (2.2)	0.005
Depression	10.1 (1.5)	11.3 (2.4)	0.02
Physical functioning	74.2 (22.3)	73.5 (17.3)	0.87
Role physical	46.1 (42.8)	44.3 (39.4)	0.83
Bodily pain	48.7 (13.7)	49.9 (11.9)	0.66
General health	63.6 (16.7)	50.3 (17.2)	0.001
Vitality	47.1 (20.3)	51.2 (13.3)	0.20
Social functioning	63.4 (14.9)	57.2 (20.8)	0.16
Role emotional	46.1 (49)	38.8 (34.7)	0.40
Mental health	57.0 (20.2)	53.2 (14.7)	0.03

*Derived from independent two samples *t*-test

## Discussion

The purpose of this study was to determine the impact of recurrent miscarriage on the quality of life and psychological distress in women with recurrent miscarriage compared to other women without history of miscarriage. The results showed that women with a history of recurrent pregnancy loss differed from women without a history of recurrent pregnancy loss on most health-related quality of life measures. The quality of life scores based on the SF-36 indicated that general health perceptions, vitality, role physical, role emotional, social functioning and mental health in women with recurrent miscarriage were lower than those without a history of multiple miscarriages. However, no significant difference was found in terms of physical functioning and bodily pain between women with and without history of recurrent miscarriage,

The finding suggests that, in order to prevent the loss of quality of life in women subsequent to a miscarriage, supportive measures should be initiated by the treatment groups to promote mental health, in addition to physical illness.

Couto et al., [10] reported that women with recurrent miscarriage had poorer results in all items including physical functioning, social functioning and role emotional, bodily pain, general health, mental health and vitality. However, in the present study, there were no significant differences between two groups in physical function and bodily pain. This difference could be due to the different status of the participants in the study because both groups of participants in mentioned study had been pregnant that due to the fear of the occurrence of abortion, their physical function has been limited and physical bodies have been created physical pain such as headaches that can be signs of anxiety. Another study showed that women with recurrent abortions had a low score in mental health and physical health [11].

In the present study, the findings indicated that individuals with recurrent miscarriage experienced more anxiety than those without a history of recurrent miscarriage. Similarly, Couto et al. [9] using the HADS found that anxiety level in women with a history of unsuccessful pregnancy was higher than that of control group. Other studies showed that two or more miscarriages were correlated with higher levels of state anxiety during pregnancy [12].

It has been reported that women with a history of miscarriage showed higher levels pregnancy-related anxiety, but studies cannot consistently suggested whether these psychological disorders remain via the subsequent pregnancy [2, 5, 13].

Mevorach-Zussman et al. [11] found that all women with recurrent miscarriage showed mild to moderate anxiety levels and these results indicate that women after experiencing recurrent miscarriage, if they do not undergo a new pregnancy or women who were pregnant subsequent to a miscarriage, may suffer higher rate of anxiety in comparison with those without such experiences. High levels of anxiety can lead to a decreased quality of life and perhaps can be a factor for spontaneous abortion in subsequent pregnancies or premature birth.

In the present study, the rate of depression increased more markedly in recurrent miscarriage group, as compared to that of the control group. Couto et al., reported that the rate of depression in pregnant women with previous adverse pregnancy was more than that of control group women which is consistent with our study results [10]. Blackmore et al., [14] found that previous recurrent pregnancy loss might be a predictor of perinatal depression. Depression not only can harden patients’ living conditions but also affects the quality of life by reducing vitality, mental health, general health perceptions and social role functioning. Therefore, the quality of life of these patients may be improved by eliminating depression.

Our findings suggested that the level of anxiety and depression of the women without children suffering from recurrent miscarriage increased more markedly, as compared to that of women with at least one child. This is probably due to their fear that they may never have children, indicating the effect of anxiety and depression on the mental health and quality of life.

If a woman has a recurrent abortion, the quality of life of the affected woman decreases, while having a child may increase mental health and general health perceptions than other women with recurrent miscarriage. It has been reported that the children-to-pregnancies ratio showed a significant associate with the mental health and sleep quality [11].

### Limitations

The descriptive nature of the study could be regarded as a limitation. In addition we did not collect data on many confounding factors that might influence the results. For instance we did not collect data on socioeconomic backgrounds of women or data on some important reproductive information including gestational age at miscarriage, and history of infertility. For future studies a better study design and a thorough collection of data are recommended.

## Conclusion

The findings from this study indicated that women with recurrent miscarriage suffer from sub-optimal health-related quality of life and experience higher level of anxiety and depression as compared to women without history of miscarriage. The findings have some implications for prenatal care delivery.

## Abbreviations

HADS: Hospital Anxiety and Depression Scale
QOL: Quality of life
SD: Standard Deviation
SF-36: Short Form Health Survey
